# Frequency of Additional Congenital Dental Anomalies in Children with Cleft Lip, Alveolar and Palate

**DOI:** 10.3390/jcm9123813

**Published:** 2020-11-25

**Authors:** Przemysław Pastuszak, Izabella Dunin-Wilczyńska, Agnieszka Lasota

**Affiliations:** Chair and Department of Jaw Orthopedics, Medical University of Lublin, 20-093 Lublin, Poland; przemyslawpastuszak@umlub.pl (P.P.); izabella.dw@umlub.pl (I.D.-W.)

**Keywords:** dentofacial deformity, orthodontics, cleft, bone graft

## Abstract

The aim of the study was to assess the risk of incidence of additional congenital dental anomalies in children with nonsyndromic cleft lip, alveolar and/without palate (CL/P). Hypodontia, hyperdontia and canines impaction was recognized. From patients with CL/P treated at the Clinic of Congenital Facial Deformities in Chair and Department of Jaw Orthopedics Medical University of Lublin, 56 subjects were randomly selected. On the panoramic radiographs taken at the age of 8–12 years, the angle of upper unerupted canines was measured using Westerlund’s recommendations. The supernumerary teeth and hypodontia were checked. The procedures: maxillary expansion, secondary alveolar bone graft (SABG) and extraction of primary canines were noted. The frequency of canines impaction was 5.36%. Hypodontia was found in 37.5% of patients; hyperdontia was present in 23.21% of patients. No influence of procedures (expansion of the maxilla, SABG, deciduous canines extraction) on permanent maxillary canine eruption was proved. Patients with CL/P are exposed to the unfavorable position of unerupted maxillary canines most frequently in the cleft area of complete cleft. Maxillary lateral incisor on the cleft side is most frequently affected with congenital anomaly. Hypodontia and hyperdontia do not influence maxillary canine impaction. Good clinical result was achieved with an applied approach, which should be widely introduced.

## 1. Introduction

Cleft lip, alveolar and/without palate (CL/P) belong to the most frequent congenital defects, occurring in approximately 10/10,000 births [1]. In these patients, dental abnormalities appear more often than in a general population, such as hypodontia, hyperdontia, incorrect teeth shape, incorrect position of the unerupted tooth in the bone, and increased risk of maxillary canines impaction.

In the descriptions of cleft complications, an increased frequency of upper canines impaction is emphasized [2,3,4]. Disturbed anatomy or genetic predispositions may lead to the movement of tooth germs at the time of eruption, and as a consequence, to the disturbed position of those teeth in the dental arch or to their complete impaction. It can cause an additional problem for the patients who, as a part of a multi-specialist treatment plan, undergo numerous surgical and orthodontic procedures. Therefore, it seems purposeful to search for factors influencing the occurrence of this abnormality or its lack.

Secondary alveolar bone grafting is one of the procedures aiming at, among others, the improvement of physiological tooth eruption in the cleft area. It was introduced by Boyne and Sands [5] and may be conducted at different ages of the patient. An early secondary bone grafting improves eruption of an upper lateral incisor and canine, and it is the most frequently planned at the age of 5–7 years [6,7]. A late secondary bone grafting tends to be performed at the age of 9–11 years when the unerupted maxillary canine is as long as 1/4–2/3, and its purpose is to create favorable conditions for permanent upper canine eruption in the cleft area [6,8].

Upper canine eruption disorder may have its source in the lack of space for those teeth caused by maxillary narrowing. In untreated occlusion in children with cleft, especially with complete bilateral cleft, the incisive bone has a tendency to forward movement, and side maxilla segments close to each other at the time of eruption, what consequently causes maxillary narrowing, particularly in the front part [9]. Similarly, in the case of unilateral clefts, maxillary narrowing is greater in the front part of an arch due to rotation of the palatal segments. This phenomenon can be assigned to mandible discontinuity and astringent effect of scars after early surgical procedures [10]. Therefore, it seems that orthodontic maxillary expansion improves canines position by ensuring the correct shape of dental arches and sufficient space for erupting teeth.

The subject matter also included an initial unfavorable position of the upper canine germ in the bone. Westerlund et al. [4] stated 10 times bigger risk of impacted canine if the inclination of the long axis of the canine germ to the mid-line is greater than 30°.

In the general population, there exists a positive correlation between the occurrence of dental anomalies such as hypodontia or reduced size of maxillary lateral incisors and canines impaction [11]. This dependency is explained by a genetic basis of the disorder or by a lack of a correctly positioned lateral incisor root adjacent to a crown of erupting canine. In the case of patients with cleft, both hypotheses can also be correct. At the same time, an interesting is an influence of supernumerary lateral incisors occurring with the frequency of 5.1–22.1% in the patients with cleft lip, alveolar cleft and cleft palate on canines impaction [12,13,14,15,16].

An aim of the work was defining the frequency of additional dental congenital anomalies in the patients with nonsyndromic cleft lip, alveolar cleft and/without cleft palate, treated at the Clinic of Congenital Facial Deformities in Chair and Department of Jaw Orthopedics Medical University of Lublin, and discovering the factors influencing the risk of canines impaction in the study group. Additionally, the frequency of hypodontia and hyperdontia occurrence was examined.

## 2. Experimental Section

From all the patients with CL/P treated at the Clinic of Congenital Facial Deformities in the Medical University of Lublin, a group of 56 persons was randomly selected. The study included persons with cleft lip, alveolar cleft and/without cleft palate—unilateral or bilateral. Patients with isolated cleft lip or cleft palate were excluded from the study. The research was conducted through the evaluation of medical documentation (patient records and X-rays).

**Figure 1:**
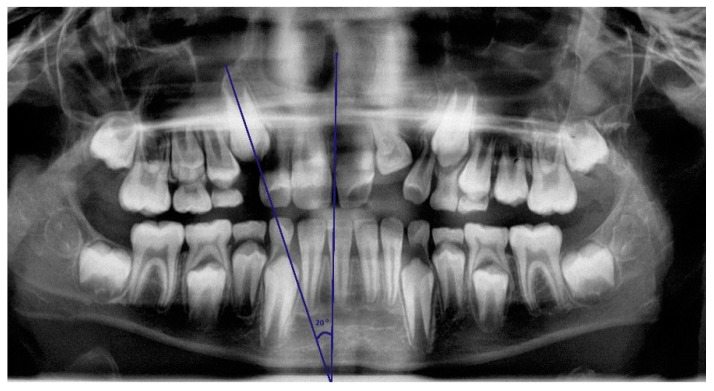
Angle of unerupted canine inclination was drawn through the long axis of the permanent maxillary canine and midsagittal vertical reference line.

On panoramic radiographs taken at the age of 8–10 years, inclination angles of the unerupted maxillary canines were measured according to Westerlund’s recommendations. (Figure 1). The measurement was taken in the Ortomed Evo software with the use of a digital protractor tool between a long axis of the permanent unerupted canine and a vertical reference line within the median plane. The vertical line was drawn based on anatomical structures, such as the nasal septum and the median suture of the maxilla and mandible. The condition for the measurement was the presence of at least 1/3 root length. The angle was measured with an accuracy of 1°, and the measurement was taken by a trained researcher. Unerupted canines with an angle greater than 30° were qualified into the group with increased risk of impaction. Canines on both sides of the jaw were examined.

Additionally, on the panoramic radiographs, the presence of supernumerary teeth and hypodontia were checked. The third molars were not taken into account.

Next, the medical documentation was evaluated after the end of the treatment, paying attention to the date of eruption of the upper permanent canines and to conducted orthodontic and surgical procedures, such as jaw expansion, secondary alveolar bone grafting and deciduous canines extraction.

The obtained results were statistically analyzed. The values of the analyzed parameters were shown with the use of count and percentage. Chi-squared test with Yates correction was used to find the occurrence of dependence between the analyzed variables. The significance level of *p* < 0.05 was settled, indicating the presence of statistically significant differences or dependencies. Database and statistical studies were conducted on the basis of the computer software Statistica 9.1 (StatSoft, Kraków, Poland).

## 3. Results

**Figure 2:**
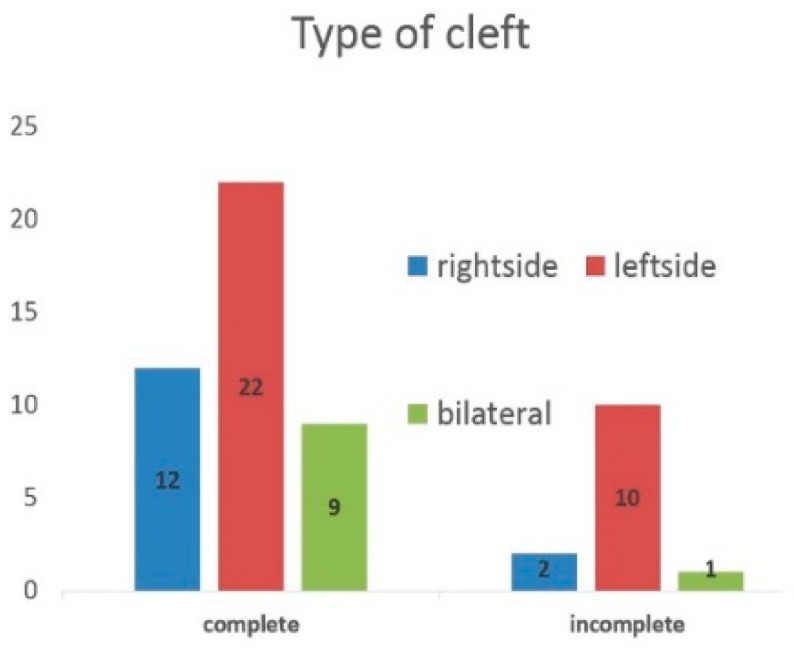
Type of cleft in the study group.

In the study group, the biggest number of patients had left side complete cleft lip, alveolar and palate. Furthermore, partial clefts more often occurred on the left side of the face (Figure 2). In almost half of respondents, unerupted canines on the side of cleft had a greater angle than 30° in relation to a reference line; that is, they had an increased risk of impaction.

Complete cleft, in comparison to partial cleft, meant a greater probability of incorrect canine angle occurrence (Table 1 and Table 2); also more often observed were dental anomalies, such as hypodontia or hyperdontia of permanent teeth. However, these differences were not statistically significant (Table 3). Therefore, the mentioned anomalies did not influence the unerupted canine position.

The frequency of canines impaction in the studied group was equal to 5.36%. When canines impaction occurred, it was always present on the cleft side (in one case, it was a partial cleft). All the impacted teeth initially had an unfavorable position angle.

Hypodontia was found in 21 (37.5%) patients, the most often regarded maxillary lateral incisors (69.44%) and mostly occurred on the cleft side. The second most frequently missing tooth was the maxillary second premolar, and the next—the mandibular second premolar (Table 4). Hyperdontia was present in 13 (23.21%) patients, and it always regarded maxillary lateral incisor, and it more often occurred on the cleft side (Table 4). Statistical analysis in the study group did not show any significant relationships between canines eruption and hypodontia, hyperdontia of permanent teeth, as well as cleft type (*p* > 0.05) (Table 5).

Late secondary alveolar bone grafting was conducted in 64.29% (36) of studied patients with an average age of 9 years 11 months. Expansion of the maxilla was performed in 53.57% (30) patients. In 32.14%, a deciduous canine tooth was extracted due to lack of physiological resorption of the root. No statistically significant influence of those procedures on permanent maxillary canine eruption was proved (Table 5).

## 4. Discussion

The frequency of canines impaction in patients with CL/P is determined in the literature as between 0% and 58% [3,16,17,18,19,20]. Lower frequency of impaction was observed in the groups above 50 persons (0–35%), while the results up to 58% were obtained in the groups with less than 50 patients [21,22,23,24]. It may indicate the higher success of specialist care in multidisciplinary centers. At the Clinic of Congenital Facial Deformities in Chair and Department of Jaw Orthopedics Medical University of Lublin, 5.36% of the studied patients impacted canine was found, which is very low frequency compared to other studies.

Akcam et al. [17] from the Ankara University, Turkey, in the study of dental anomalies among 122 patients with cleft, found out that the least frequently impacted canines occurred in patients with unilateral cleft on the healthy side—3.8%, while the most frequently, mostly up to 25.7%, in bilateral cleft.

According to much research, the unfavorable inclination of the canine increased the probability of maxillary canines impaction [2,3]. Westerlund et al. [4] determined a limit angle at 30°. In her study, the most unfavorable position of the unerupted canine was present on the cleft side—averagely 31.9° at the age of 10 years, as compared to the healthy side—15.6°. This result is consistent with our study, where 87% of unerupted canines with an angle over 30° were present on the cleft side. In opposition to these studies is the statement of Vellone et al. [25], who claims that the inclination of the canine does not significantly influence its impaction, although the research was conducted on a sample of only 24 patients. In our own study, 44.64% of patients had an incorrect unerupted canine angle. However, in spite of this, in 94.64% of them, the teeth erupted spontaneously. No statistically significant impact of any of the medical procedures on this result was found. Therefore it seems that the general scheme of conduction and all the multidisciplinary procedures connected with each other can significantly influence the low percentage of impacted canines.

The impact of secondary bone grafting on the process of maxillary canines eruption in patients with a cleft is a subject of numerous discussions. Enemark et al. [26] analyzed 62 cases with unilateral complete cleft with an average patient’s age of 12 years at the time of the procedure and proved that 5 unerupted canines changed their path of eruption into unfavorable after the secondary bone grafting. However late age of patients needs to be taken into consideration. A high percentage of erupted teeth was found in the study of Trindade et al. [27] regarding 65 persons with unilateral cleft (UCLP), who at the age of 9–12 years underwent the secondary bone grafting procedure. The frequency of spontaneous eruption in this study increases together with time from the procedure, from 47% after 2 years, through 72% after 3 years, up to 95% after 4 years. In the study of Kumar et al. [28] conducted in the centers in Australia and in the UK, among 56 persons with unilateral or bilateral cleft, 95% of canines erupted up to one year after the procedure. Matsui et al. [29] performed the secondary alveolar bone grafting in 190 patients with unilateral cleft lip/palate and unerupted canines, in the average age of 9 years 1 month, and in this group, 78.9% of canines erupted spontaneously. In the own study, in 94.44% of cases after the secondary bone grafting (average age of 9 years 11 months), the canines erupted spontaneously, although no statistical significance of this procedure’s influence on canines eruption was proved.

In patients with cleft, hypodontia occurs statistically more often than in the general population—from 4.4% to 13.4% [30], while in the patients with cleft lip, alveolar cleft and/or cleft palate, it amounts from 25% [31]) to 77% [32]. In the studied material, tooth bud agenesis was found in 37.5% of persons. Similar to data from the literature, the most frequently missing tooth was the maxillary lateral incisor [32,33,34]. Hypodontia was more often present on the cleft side (69.44%) and more frequently concerned complete clefts (80.55%). Furthermore, the study of Shapira et al. [32] showed the more frequent occurrence of hypodontia on the cleft side—92% of missing teeth on the cleft side, while no significant differences between partial and complete clefts were found. Taking into consideration the general population, where the most frequently missing tooth is the second premolar, the most often lack of maxillary lateral incisor in patients with cleft may signify the genetic background. The dependency was found between the MSX1 as well as PAX9 gene mutations and the occurrence of tooth bud agenesis in patients with cleft [35]. In the studied material, no statistically significant relationship was found between the presence of hypodontia and the frequency of impacted maxillary canines in persons with a cleft. The same results were obtained by Tortora et al. [4] Oberoi et al. [36], Kleinpoort et al. [7]. However, there exist studies indicating an increased risk of maxillary canine impaction in the case of lack of lateral incisor [2,37].

Another dental anomaly with significantly greater frequency present in patients with CL/P is hyperdontia. It seems that supernumerary teeth may influence the deterioration of the conditions for the eruption of permanent canines. In the study group, the supernumerary teeth were present in 23.21% of patients. A similar frequency of the anomaly was proved by da Silva et al. [38]—29% and Tan et al. [34]—21.7%. In both cases, similarly to our study, the most frequently supernumerary tooth was maxillary lateral incisor, mostly present on the cleft side. In the present study, none of the persons with impacted canines had a supernumerary lateral incisor, although the result was not statistically significant. However, there exist reports that all the anomalies regarding lateral incisors, including their hyperdontia, increase the risk of canine impaction [2].

Due to the low number of impacted canines in our patients, no statistically significant results (*p* > 0.05) indicating the influence of particular procedures (expansion of the maxilla, secondary alveolar bone grafting, unresorbed deciduous canine extraction) on impaction frequency reduction were found. A low number of unerupted canines in patients with cleft lip, alveolar cleft and cleft palate at the Clinic of Congenital Facial Deformities at the Medical University of Lublin, compared to other units in the world, may be influenced by total orthodontic care that is offered to persons with cleft in Poland. The program ensures free orthodontic and surgical treatment from birth until adulthood. Patients are often and regularly seen at the control check-ups, which allows for the implementation of appropriate procedures at the best possible time.

## 5. Conclusions

Patients with cleft are exposed to an unfavorable position of unerupted maxillary canines. Incorrect unerupted maxillary canine angle the most frequently occurs in the cleft area and more often concerns patients with complete cleft.

The most frequently quantitatively disturbed tooth in patients with cleft is maxillary lateral incisor on the cleft side—when it is both missing and supernumerary.

Hypodontia and hyperdontia do not influence the frequency of maxillary canines impaction in patients with cleft.

Although no influence of particular procedures on impaction frequency reduction was found, good clinical result was achieved with a total specialistic multidisciplinary approach which can be introduced in other cleft centers.

## Figures and Tables

**Table 1:** Relationship between a type of cleft and unerupted upper canine angulation.

Cleft	Unerupted Canine Angle in the Maxilla		Total
	Correct	Incorrect	
Complete	56	30 *	86
	65.12%	34.88% *	
Incomplete	25	1 *	26
	96.15%	3.85% *	
	Chi^2^_Y_ = 8.120, *p* = 0.004		

* Statistically significant differences between groups (*p* < 0.05).

**Table 2:** Relationship between a type of cleft and unerupted canine angulation on the cleft side.

Cleft	Unerupted Canine Angle on the Cleft Side		Total
	Correct	Incorrect	
Complete	26	26 *	52
	50%	50% *	
Incomplete	13	1 *	14
	92.86%	7.14% *	
	Chi^2^_Y_ = 6.702, *p* = 0.010		

* Statistically significant differences between groups (*p* < 0.05).

**Table 3:** Relationship between a type of cleft, unerupted canine angulation and hypodontia or hyperdontia.

Analyzed Variable		Hypodontia		Total	*p* Value	Hyperdontia		Total	*p* Value
		+	−			+	−		
Cleft	complete	17	26	43	*p* = 0.806	11	32	43	*p* = 0.698
	incomplete	4	9	13		2	11	13	
Unerupted canine angle	correct	13	18	31	*p* = 0.627	6	25	31	*p* = 0.657

**Table 4:** Hypodontia and hyperdontia in various types of cleft.

Type of Cleft			Left Side Cleft Lip and Alveolar Cleft		Left Side Cleft Lip, Alveolar Cleft and Cleft Palate		Right Side Cleft Lip and Alveolar Cleft		Right Side Cleft Lip, Alveolar Cleft and Cleft Palate		Bilateral Cleft Lip and Alveolar Cleft		Bilateral Cleft Lip, Alveolar Cleft and Cleft Palate		Total
Number of Persons in the Group			*n* = 10		*n* = 22		*n* = 2		*n* = 12		*n* = 1		*n* = 9		56
Side of dental anomaly			right	left	right	left	right	left	right	left	right	left	right	left	
Hypodontia	Maxillary lateral incisor			1	5	8	1	1	4		1	1	1	2	25
	Second premolar	Maxillary			2	3					1		1		7
		Mandibular	1		2	1									4
Hyperdontia	Maxillary lateral incisor			2	2	2			3	1			2	2	14

**Table 5:** Relationship between a type of cleft, unerupted canine angle, hypodontia, hyperdontia, treatment procedures and canines eruption.

Analyzed Variable		Canines Eruption		Total	*p* Value
		+	−		
Cleft	complete	12	1	13	*p* = 0.782
		92.31%	7.69%		
	incomplete	41	2	43	
		95.35%	4.65%		
Unerupted canine angle	correct	31	0	31	*p* = 0.166
		100%	0%		
	incomplete	22	3	25	
		88%	12%		
Hyperdontia	−	40	3	43	*p* = 0.782
		93.02%	6.98%		
	+	13	0	13	
		100%	0%		
Hypodontia	−	33	2	35	*p* = 0.646
		94.29%	5.71%		
	+	20	1	21	
		95.24%	4.76%		
Expansion of the maxilla	−	24	2	26	*p* = 0.899
		92.31%	7.69%		
	+	29	1	30	
		96.67%	3.33%		
Deciduous canine extraction	−	35	2	37	*p* = 0.542
		94.59%	5.41%		
	+	17	1	18	
		94.44%	5.56%		
Secondary alveolar bone grafting	−	18	1	19	*p* = 0.563
		94.74%	5.26%		
	+	34	2	36	
		94.44%	5.56%		
